# Radiotherapy Plus Chemotherapy Leads to Prolonged Survival in Patients With Anaplastic Thyroid Cancer Compared With Radiotherapy Alone Regardless of Surgical Resection and Distant Metastasis: A Retrospective Population Study

**DOI:** 10.3389/fendo.2021.748023

**Published:** 2021-11-01

**Authors:** Weili Zhou, Yangyang Yue, Xin Zhang

**Affiliations:** ^1^ Department of General Surgery, Shengjing Hospital of China Medical University, Shenyang, China; ^2^ Department of Health Management, Shengjing Hospital of China Medical University, Shenyang, China; ^3^ Department of Radiology and Nuclear Medicine, Shengjing Hospital of China Medical University, Shenyang, China

**Keywords:** anaplastic thyroid cancer, chemotherapy, distant metastasis, radiotherapy, surgical resection, SEER database

## Abstract

**Background:**

Whether anaplastic thyroid cancer (ATC) patients benefit more from radiotherapy plus chemotherapy (RCT) than from radiotherapy alone (RT) was controversial. We aimed to investigate the effectiveness of RCT *versus* RT on ATC overall and within subgroups by surgical resection and distant metastasis in a large real-world cohort.

**Methods:**

Patients with ATC diagnosed between 2004 and 2015 were identified from the Surveillance, Epidemiology, and End Results Program database. Inverse probability weighting (IPW) was performed to balance variables between the two groups. Multivariate Cox proportional hazard model and Fine-Gray compete-risk model were carried out to investigate prognostic factors relating to overall survival (OS) and cancer-specific survival (CSS). Subgroup analysis was carried out, and a forest plot was graphed.

**Results:**

Of the 491 ATC patients, 321 (65.4%) were in the RCT group and 170 (34.6%) were in the RT group. The median OS was 4 months [interquartile range (IQR) 2–7] and 2 months (IQR 1–4) for patients in the RCT and RT groups, respectively. As indicated by the inverse probability weighting multivariate regression, RCT was associated with significantly improved OS (adjusted HR = 0.69, 95% CI = 0.56–0.85, *p* *<* 0.001) and CSS (adjusted subdistribution HR = 0.77, 95% CI = 0.61–0.96, *p* *=* 0.018). The prominent effect of RCT *versus* RT alone remains significant within each subgroup stratified by surgical resection and distant metastasis. Older age, single marital status, surgical resection, distant metastasis, and tumor extension were significant prognostic factors of survival.

**Conclusions:**

RCT contributes to prolonged OS and CSS compared with RT alone in ATC patients, regardless of surgical resection and distant metastasis. RCT should be preferentially applied to ATC patients.

## Introduction

Thyroid cancer is a rare malignant tumor that accounts for about 2.9% of all site cancer cases in the USA, with more than 52 thousand newly diagnosed cases and nearly 2,200 new deaths yearly (1). Anaplastic thyroid cancer (ATC), which accounts for less than 2% of thyroid cancer but leads to more than 50% of the annual thyroid cancer-related mortality, remains one of the most aggressive and fatal tumors. ATC augments rapidly, invades neck mass and regional lymph nodes, with a median survival of 4 months and nearly 50% of newly diagnosed ATC patients having distant metastasis (2–4).

Even though novel immunotherapy and targeted therapy, such as pembrolizumab, bevacizumab, and sorafenib, have been administered with traditional therapy using surgical resection and radiotherapy with or without chemotherapy within clinical trials, the survival outcome of ATC patients remains disappointing (5, 6). Thus, the best treatment for ATC is still suggested as surgical resection combined with radiotherapy with or without chemotherapy (6–9). However, the effect of chemotherapy administered with RT was unascertained and variable in different studies focusing on different subsets of ATC patients. For example, some small retrospective studies failed to identify a significant benefit from radiotherapy plus chemotherapy (RCT) (10, 11), while other studies proposed the potential benefit of RCT (12). The inconsistency of the effect of chemotherapy may be due to the heterogeneity of ATC patients between different treatment groups because the heterogeneity biases the results of previous studies and is hard to control due to the extreme rareness of ATC. Moreover, because of the rareness of ATC, random control trials focusing on comparing RCT with radiotherapy alone (RT) are not feasible. Furthermore, no studies carrying out subgroup analysis by surgical resection and distant metastasis have been carried out.

Most previous studies have a small sample size with the heterogeneity of patients not well adjusted. Whether ATC patients could benefit more from RCT than from RT was unclear. Thus, a study comparing RCT with RT alone in a large representative cohort that comprises diverse subsets of ATC patients is of great importance. Therefore, we carried out a study using the inverse probability weighting technique to adjust heterogeneity of patients to investigate the efficacy of RCT *versus* RT on the ATC using the Surveillance, Epidemiology, and End Results (SEER) Program database of the National Cancer Institute.

## Materials and Methods

### Data Source and Study Population

The SEER database was used to identify ATC patients diagnosed between 2004 and 2015 using the International Classification of Diseases for Oncology 3rd Edition (ICD-O-3). Patients with a primary site code of C73.9 and the ICD-O-3 histology codes of 8020–8035 were included. Patients were excluded according to the following criteria: (1) more than one malignant tumor; (2) without radiotherapy; and (3) no mass tumor found. A survival time of 0 months was recoded as 0.5 months to more accurately represent patients who died within 1 month of their diagnosis but did not reach the 1-month threshold (13).

As potential prognostic factors, year of diagnosis, race, age at diagnosis, gender, marital status, multifocality, lymph node invasion (American Joint Committee on Cancer (AJCC) N stage), distant metastasis (AJCC M stage), tumor size, surgery type, and tumor extension were derived from the corresponding fields of the SEER database (4, 14–16). The year of diagnosis was grouped into two intervals in the year 2010. The age at diagnosis was grouped into two intervals at age 65. Tumor extension codes, which indicates the continuous growth of the primary tumor, was merged into six groups, according to the definition of tumor extension codes (17): 100, 200, 300, and 400 as the group I, indicating tumors confined to the thyroid capsule; 450 and 480 as the group II, indicating minimal extra thyroid extension; 500, 520, 550, and 560 as the group III, indicating involving of parathyroid, recurrent laryngeal, vagus, cricoid cartilage, esophagus, larynx, sternocleidomastoid muscle, and trachea; 600, 620, 650, 700, 730, 800, and 810 as group IV, being equivalent to AJCC T4b stage and indicating involving of the thyroid cartilage, carotid artery (encased), jugular vein, and thyroid artery or vein, bone, skeletal muscle (other than the strap or sternocleidomastoid muscle), mediastinal tissues, and prevertebral fascia; 815 as group V, being equivalent to AJCC T4 NOS; and 999 as group unknown.

The detailed information about the RT protocols, chemotherapy agents, sequence of RT or RCT and surgery, immunotherapy, targeted therapy, the specific drugs and doses used are not available in the SEER database; thus, these factors could not be evaluated and controlled in this study.

### Outcomes

Overall survival (OS) was the primary outcome. ATC-specific survival (CSS) was the secondary outcome, with death attributable to reasons other than ATC being considered compete risk.

### Statistical Analysis

The stabilized inverse probability weighting (SIPW) was applied to balance variables between groups (18). All the variables available in this study were included in logit regression models to calculate the probability of receiving RCT *versus* RT. SIPW weights were then calculated based on the precalculated logit models. We also calculated SIPW weights within each subgroup stratified by surgical resection and distant metastasis.

The Kaplan-Meier survival curves were plotted and compared by the Cox test due to application of IPW adjustment. Multivariate Cox proportional hazard regression models and Fine-Gray compete-risk models were applied to calculate the (subdistribution) hazard ratio [(s)HR] and their corresponding 95% confidence intervals (95% CIs). Univariate regression models were not performed. All the variables were included in a multivariate model without variable filtering because filtering variables from univariate regression basing on *p*-value is controversial.

In order to adjust for and minimize the potential immortal-time bias, the conditional landmark analysis was carried out at cutoffs of 1, 2, and 3 months because patients who were prescribed to receive RT or RCT need sufficient time after diagnosis to get the corresponding therapy (19, 20).

A two-tailed *p-*value of less than 0.05 was considered statistically significant. All the statistical processes were performed in the STATA 16.0 software (StataCorp, College Station, TX, USA).

## Results

### Baseline Characteristics

**Figure 1:**
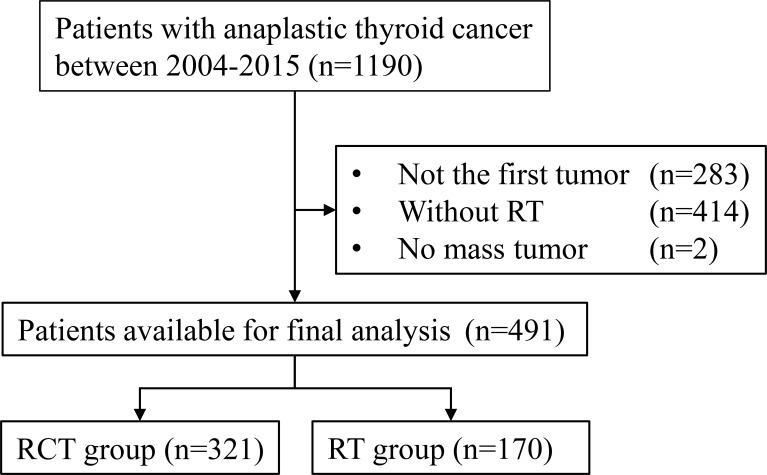
Flowchart of the patient selection procedure. RCT, radiotherapy plus chemotherapy; RT, radiotherapy alone.

The sample selection procedure is shown in **Figure 1**. Of the 491 patients available for the final analysis, 321 (65.4%) were in the RCT group and 170 (34.6%) were in the RT group. The median follow-up of patients in the RCT and RT groups was 5 (interquartile range (IQR) 3–12) and 3 months (IQR 1–6), respectively. The SIPW-adjusted median OS was 4 (IQR 2–7) and 2 months (IQR 1–4) for patients in the RCT and RT groups. The RCT group had more patients aged less than 65 (49.5% *vs*. 29.40%, *p* < 0.001). More patients were male in the RCT group than in the RT group (46.7% *vs*. 37.1%, *p* = 0.04). In the RCT group, there were more patients with solitary tumor (67.0% *vs*. 63.5%, *p* = 0.008) and more patients without distant metastasis (57.3% *vs*. 42.4%, *p* = 0.007). Less patients in the RCT group received no surgery (48.0% *vs*. 60.6%, *p* = 0.024) (**Table 1**).

**Table 1:** Baseline characteristics.

Characteristics	RT	RCT	*p*-value
	*N* (%)	*N* (%)	
Year of diagnosis			
2004–2009	84 (49.4)	144 (44.9)	0.336
2010–2015	86 (50.6)	177 (55.1)	
Race			
Black	14 (8.2)	22 (6.9)	0.245
White	135 (79.4)	273 (85.0)	
Other	21 (12.4)	26 (8.1)	
Age (year)			
<65	50 (29.4)	159 (49.5)	<0.001
≥65	120 (70.6)	162 (50.5)	
Gender			
Female	107 (62.9)	171 (53.3)	0.04
Male	63 (37.1)	150 (46.7)	
**Marital Status**			0.086
Married	94 (55.3)	200 (62.3)	
Single	15 (8.8)	40 (12.5)	
Divorced/separated/widowed	54 (31.8)	72 (22.4)	
Unknown	7 (4.1)	9 (2.8)	
Multifocality			
Solitary	108 (63.5)	215 (67.0)	0.008
Multifocal	21 (12.4)	61 (19.0)	
Unknown	41 (24.1)	45 (14.0)	
Lymph nodes invasion			
N0	55 (32.4)	116 (36.1)	0.595
N1NOS	17 (10.0)	26 (8.1)	
N1a	16 (9.4)	41 (12.8)	
N1b	59 (34.7)	102 (31.8)	
NX	23 (13.5)	36 (11.2)	
Distant metastasis			
M0	72 (42.4)	184 (57.3)	0.007
M1	87 (51.2)	121 (37.7)	
MX	11 (6.5)	16 (5.0)	
Tumor size (cm)			
<2	4 (2.4)	3 (0.9)	0.476
2–4	18 (10.6)	37 (11.5)	
≥4	114 (67.1)	227 (70.7)	
Unknown	34 (20.0)	54 (16.8)	
Surgery			
No	103 (60.6)	154 (48.0)	0.024
Nonthyroidectomy	26 (15.3)	57 (17.8)	
Thyroidectomy	41 (24.1)	110 (34.3)	
Tumor extension			
I	26 (15.3)	44 (13.7)	0.188
II	15 (8.8)	24 (7.5)	
III	42 (24.7)	74 (23.1)	
IV	66 (38.8)	157 (48.9)	
V	3 (1.8)	5 (1.6)	
VI	18 (10.6)	17 (5.3)	
Outcome			
Alive	12 (7.1)	40 (12.5)	0.302
Dead of other causes	7 (4.1)	10 (3.1)	
Dead attributable to ATC	148 (87.1)	266 (82.9)	
Dead of unknown cause	3 (1.8)	5 (1.6)	

RCT, radiotherapy plus chemotherapy; RT, radiotherapy alone; cm, centimeter; ATC, anaplastic thyroid cancer.

### Prognostic Factors Associated With Overall Survival and Cancer-Specific Survival

As shown in **Table 2**, based on the multivariate Cox model, patients who underwent RCT had significantly improved OS than those who underwent RT alone both before (unadjusted sHR = 0.69, 95% CI = 0.56–0.84, *p* *<* 0.001) and after the SIPW (adjusted HR = 0.69, 95% CI = 0.56–0.85, *p* *<* 0.001). After the SIPW, older age (adjusted HR = 1.31, 95% CI = 1.07–1.62, *p* *=* 0.011), single marital status (adjusted HR = 1.67, 95% CI = 1.21–2.30, *p* *=* 0.002), distant metastasis (adjusted HR = 1.87, 95% CI = 1.52–2.30, *p* *<* 0.001), and more aggressive tumor extension (reference: group I; group IV: adjusted HR = 1.64, 95% CI = 1.17–2.30, *p* = 0.004) were all significant negative prognostic factors of OS. While surgical resection (reference: No; Nonthyroidectomy: adjusted HR = 0.68, 95% CI = 0.53–0.89, *p* *=* 0.004; Thyroidectomy: adjusted HR = 0.51, 95% CI = 0.40–0.66, *p* *<* 0.001) was a significant positive prognostic factors of OS. Moreover, year of diagnosis, race, gender, multifocality, lymph nodes invasion, and tumor size were not significant prognostic factors of OS (*p* all >0.05).

**Table 2:** Multivariate Cox proportional hazard model of overall survival before and after inverse-probability weighting.

Characteristics	Origin cohort		IPW cohort	
	Unadjusted HR (95% CI)	*p*-value	Adjusted HR (95% CI)	*p*-value
Treatment				
RT	Reference		Reference	
RCT	0.69 (0.56–0.84)	<0.001	0.69 (0.56–0.85)	<0.001
Year of diagnosis				
2004–2009	Reference		Reference	
2010–2015	1.01 (0.84–1.21)	0.936	1.00 (0.82–1.21)	0.962
Race				
White	Reference		Reference	
Black	0.90 (0.67–1.21)	0.483	0.86 (0.64–1.17)	0.336
Other	1.11 (0.76–1.63)	0.575	1.11 (0.76–1.63)	0.578
Age (year)				
<65	Reference		Reference	
≥65	1.29 (1.06–1.57)	0.010	1.31 (1.07–1.62)	0.011
Gender				
Female	Reference		Reference	
Male	1.10 (0.92–1.32)	0.297	1.08 (0.89–1.32)	0.427
Marital status				
Married	Reference		Reference	
Single	1.56 (1.16–2.11)	0.003	1.67 (1.21–2.30)	0.002
Divorced/separated/widowed	1.15 (0.92–1.45)	0.228	1.16 (0.91–1.47)	0.238
Unknown	0.79 (0.45–1.38)	0.413	0.79 (0.45–1.38)	0.411
Multifocality				
Solitary	Reference		Reference	
Multifocal	0.80 (0.60–1.06)	0.118	0.79 (0.57–1.08)	0.143
Unknown	1.17 (0.93–1.46)	0.184	1.07 (0.85–1.35)	0.545
Lymph nodes invasion				
N0	Reference		Reference	
N1NOS	1.13 (0.84–1.52)	0.422	1.09 (0.79–1.49)	0.605
N1a	0.90 (0.65–1.24)	0.519	0.92 (0.66–1.27)	0.603
N1b	1.08 (0.86–1.37)	0.499	1.09 (0.84–1.40)	0.523
NX	0.86 (0.61–1.20)	0.375	0.82 (0.59–1.15)	0.248
Distant metastasis				
M0	Reference		Reference	
M1	1.88 (1.53–2.29)	<0.001	1.87 (1.52–2.30)	<0.001
MX	1.42 (0.93–2.17)	0.100	1.33 (0.83–2.13)	0.230
Tumor size (cm)				
<2	Reference		Reference	
2–4	0.77 (0.42–1.44)	0.418	0.78 (0.44–1.36)	0.379
≥4	0.94 (0.54–1.65)	0.830	0.95 (0.57–1.56)	0.835
Unknown	1.43 (0.78–2.64)	0.245	1.50 (0.87–2.58)	0.145
Surgery				
No	Reference		Reference	
Nonthyroidectomy	0.65 (0.51–0.83)	0.001	0.68 (0.53–0.89)	0.004
Thyroidectomy	0.52 (0.41–0.66)	<0.001	0.51 (0.40–0.66)	<0.001
Tumor extension				
I	Reference		Reference	
II	1.32 (0.87–2.02)	0.193	1.28 (0.81–2.01)	0.289
III	1.22 (0.88–1.70)	0.239	1.24 (0.87–1.78)	0.233
IV	1.60 (1.17–2.20)	0.003	1.64 (1.17–2.30)	0.004
V	1.42 (0.86–2.34)	0.169	1.39 (0.81–2.38)	0.236
VI	1.00 (0.64–1.58)	0.989	1.00 (0.61–1.62)	0.988

HR, hazard ratio; IPW, inverse-probability weighting; RCT, radiotherapy plus chemotherapy; RT, radiotherapy alone; cm, centimeter.

Similarly, in the multivariate CR model, patients who underwent RCT had improved CSS both before (unadjusted sHR = 0.75, 95% CI = 0.60–0.93, *p* *=* 0.008) and after the SIPW (adjusted sHR = 0.77, 95% CI = 0.61–0.96, *p* *=* 0.018) compared with those who underwent RT. After the SIPW, distant metastasis (adjusted sHR = 1.93, 95% CI = 1.54–2.41, *p* *<* 0.001), and more aggressive tumor extension (reference: group I; group IV: adjusted sHR = 1.73, 95% CI = 1.21–2.47, *p* = 0.003; group V: adjusted sHR = 1.78, 95% CI = 1.04–3.06, *p* = 0.035) were significant negative prognostic factors of CSS, while surgery (reference: No; Nonthyroidectomy: adjusted sHR = 0.66, 95% CI = 0.48–0.90, *p* = 0.009; thyroidectomy: adjusted sHR = 0.54, 95% CI = 0.42–0.70, *p* < 0.001) were significant positive prognostic factors of CSS. Furthermore, the rest factors were also not significant prognostic factors of CSS (*p* all >0.05) (**Table 3**).

**Table 3:** Multivariate Fine-Gray compete-risk model of cancer-specific survival before and after inverse-probability weighting.

Characteristics	Origin cohort		IPW cohort		
	Unadjusted sHR (95% CI)	*p*-value	Adjusted sHR (95% CI)	*p*-value	
Treatment					
RT	Reference		Reference		
RCT	0.75 (0.60–0.93)	0.008	0.77 (0.61–0.96)		0.018
Year of diagnosis					
2004–2009	Reference		Reference		
2010–2015	0.91 (0.74–1.12)	0.368	0.88 (0.71–1.08)		0.22
Race					
White	Reference		Reference		
Black	0.91 (0.68–1.23)	0.558	0.91 (0.66–1.25)		0.559
Other	1.14 (0.79–1.64)	0.487	1.13 (0.77–1.66)		0.536
Age (year)					
<65	Reference		Reference		
≥65	1.13 (0.92–1.39)	0.237	1.15 (0.93–1.44)		0.204
Gender					
Female	Reference		Reference		
Male	0.90 (0.73–1.10)	0.297	0.86 (0.69–1.08)		0.207
Marital status					
Married	Reference		Reference		
Single	1.21 (0.85–1.72)	0.296	1.22 (0.82–1.82)		0.333
Divorced/separated/widowed	1.13 (0.89–1.43)	0.327	1.09 (0.86–1.40)		0.47
Unknown	0.78 (0.42–1.44)	0.427	0.71 (0.37–1.38)		0.317
Multifocality					
Solitary	Reference		Reference		
Multifocal	0.95 (0.73–1.24)	0.703	0.98 (0.72–1.32)		0.872
Unknown	1.22 (0.90–1.65)	0.208	1.16 (0.87–1.53)		0.311
Lymph nodes invasion					
N0	Reference		Reference		
N1NOS	1.09 (0.78–1.52)	0.619	1.06 (0.75–1.49)		0.759
N1a	0.85 (0.60–1.19)	0.333	0.86 (0.61–1.22)		0.408
N1b	0.88 (0.69–1.12)	0.292	0.82 (0.62–1.08)		0.15
NX	0.98 (0.71–1.36)	0.919	0.92 (0.67–1.26)		0.593
Distant metastasis					
M0	Reference		Reference		
M1	1.97 (1.60–2.44)	<0.001	1.93 (1.54–2.41)		<0.001
MX	1.39 (0.89–2.19)	0.151	1.31 (0.78–2.19)		0.306
Tumor size (cm)					
<2	Reference		Reference		
2–4	1.01 (0.53–1.95)	0.967	1.05 (0.58–1.93)		0.863
≥4	1.18 (0.64–2.16)	0.603	1.20 (0.68–2.10)		0.526
Unknown	1.48 (0.77–2.86)	0.241	1.56 (0.84–2.88)		0.156
Surgery					
No	Reference		Reference		
Nonthyroidectomy	0.67 (0.51–0.88)	0.003	0.66 (0.48–0.90)		0.009
Thyroidectomy	0.57 (0.46–0.73)	<0.001	0.54 (0.42–0.70)		<0.001
Tumor extension					
I	Reference		Reference		
II	1.52 (0.96–2.38)	0.072	1.47 (0.88–2.44)		0.141
III	1.34 (0.95–1.89)	0.098	1.34 (0.92–1.96)		0.126
IV	1.66 (1.19–2.31)	0.003	1.73 (1.21–2.47)		0.003
V	1.77 (1.08–2.90)	0.025	1.78 (1.04–3.06)		0.035
VI	1.15 (0.73–1.81)	0.555	1.12 (0.69–1.83)		0.642

sHR, subdistribution hazard ratio; IPW, inverse-probability weighting; RCT, radiotherapy plus chemotherapy; RT, radiotherapy alone; cm, centimeter.

**Figure 2:**
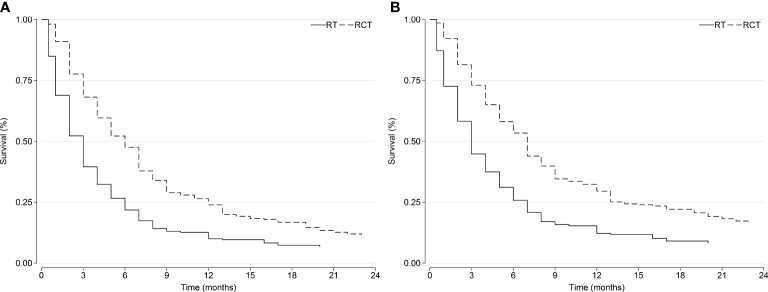
Overall survival and cancer-specific survival curves after the inverse probability weighting: **(A)** overall survival and **(B)** cancer-specific survival. RCT, radiotherapy plus chemotherapy; RT, radiotherapy alone.

The SIPW adjusted Kaplan-Meier survival curves of CSS and OS are illustrated in **Figure 2**. Patients in the RCT group survived longer than those in the RT group, and the difference was statistically significant (Cox test *p* all <0.05).

### Subgroup Analysis

**Figure 3:**
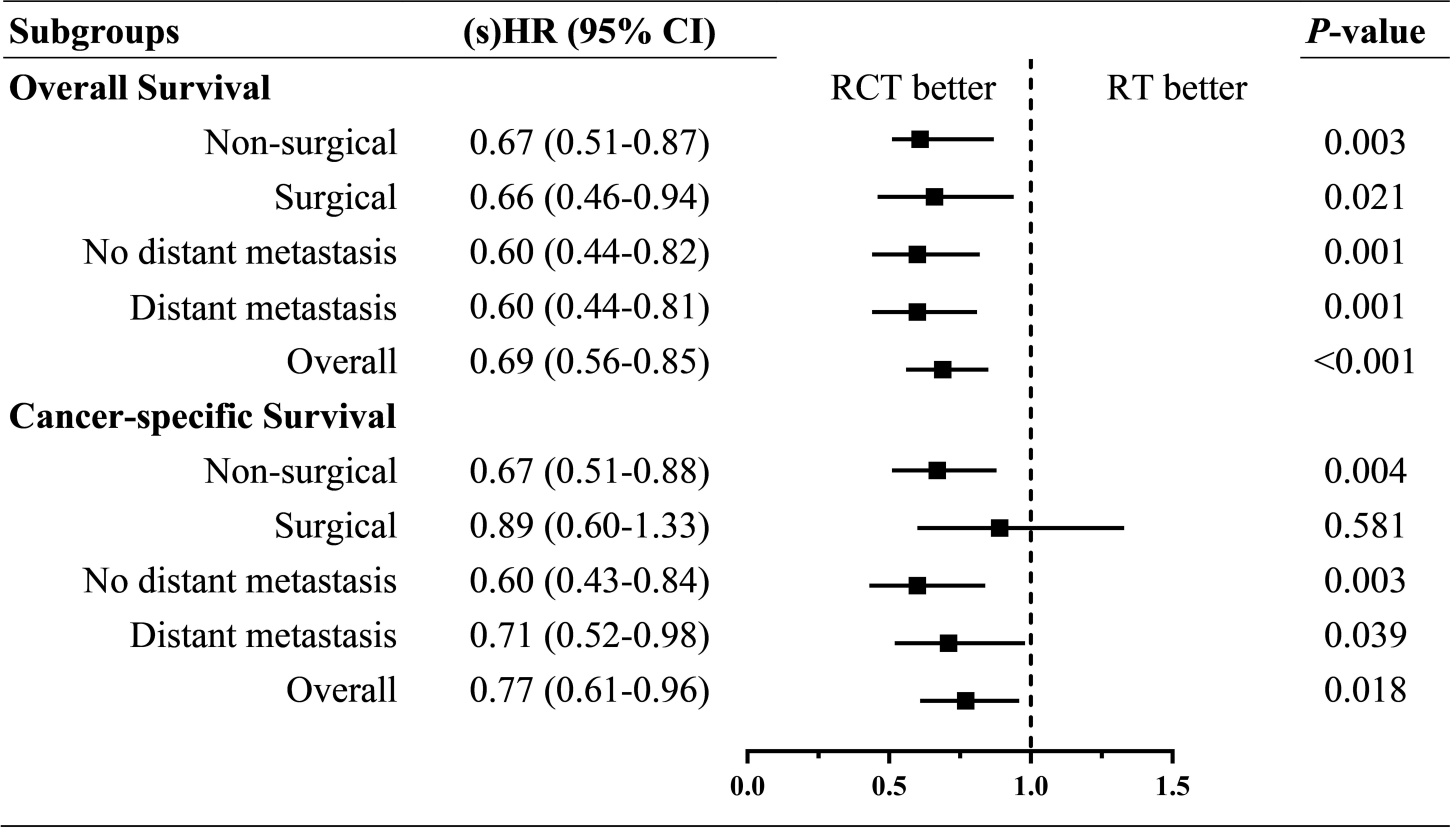
Forest plot of (sub distribution) hazard ratios of RCT *versus* RT within each subgroup by surgical resection and distant metastasis. sHR, subdistribution hazard ratio; HR, hazard ratio.

To assess the effectiveness of RCT compared with that of RT within particular subsets of the ATC patients, we conducted SIPW-adjusted multivariate regressions for OS and CSS within each subgroup stratified by surgical resection and distant metastasis. The (s)HRs of RCT *versus* RT alone from multivariate regression within each subgroup are summarized in a forest plot (**Figure 3**). Moreover, the significant beneficial effects of RCT compared with RT alone on OS and CSS were present within all the subgroups, except for the surgical resection subgroup for CSS.

### Landmark Analysis

In the landmark analysis, the beneficial effect of RCT remained persistent but lost statistical significance within most of the subgroups, except for the consistently significant efficacy of RCT within patients having no distant metastasis (**Table 4**).

**Table 4:** Adjusted (subdistribution) hazard ratios of RCT *versus* RT overall and within each subgroup by surgical resection and distant metastasis after the inverse-probability weighting in the landmark analysis at cutoffs of 1, 2, and 3 months.

Subgroups	1 Month		2 Months		3 Months	
	*N*	Adjusted (s)HR (95% CI)	*N*	Adjusted (s)HR (95% CI)	*N*	Adjusted (s)HR (95% CI)
Overall survival						
Overall	455	0.77 (0.62–0.96)^*^	407	0.82 (0.64–1.04)	334	0.88 (0.66–1.16)
Nonsurgical	219	0.85 (0.64–1.13)	182	0.83 (0.60–1.15)	133	0.81 (0.52–1.28)
Surgical	232	0.67 (0.47–0.96)^*^	222	0.76 (0.52–1.10)	195	0.88 (0.58–1.34)
No distant metastasis	243	0.62 (0.45–0.85)^*^	230	0.66 (0.48–0.91)^*^	204	0.62 (0.44–0.88)^*^
Distant metastasis	188	0.77 (0.55–1.06)	157	0.80 (0.55–1.16)	100	1.10 (0.70–1.73)
Cancer-specific survival						
Overall	455	0.84 (0.67–1.07)	407	0.87 (0.67–1.13)	334	0.91 (0.68–1.22)
Nonsurgical	219	0.79 (0.59–1.06)	182	0.77 (0.55–1.08)	133	0.88 (0.54–1.43)
Surgical	232	0.91 (0.61–1.36)	222	0.92 (0.61–1.38)	195	0.85 (0.55–1.31)
No distant metastasis	243	0.58 (0.41–0.82)^*^	230	0.62 (0.44–0.87)^*^	204	0.59 (0.40–0.87)^*^
Distant metastasis	188	0.94 (0.67–1.33)	157	0.96 (0.64–1.43)	100	1.26 (0.77–2.06)

(s)HR, (subdistribution) hazard ratio; RCT, radiotherapy plus chemotherapy; RT, radiotherapy alone.

^*^p-value < 0.05.

## Discussion

The critical findings of this study are that RCT leads to significantly prolonged OS and CSS in ATC patients compared with RT alone, regardless of surgical resection and distant metastasis. To our knowledge, this study is the first large-scale retrospective study comprehensively comparing RCT with RT alone within different subsets of ATC patients, controlling for some factors never adjusted in previous studies, such as multifocality and tumor extension. With SIPW adjustment and subgroup analysis carried out, this study obtains robust results about the superior effectiveness of RCT *versus* RT. Our study adds to the supportive evidence of the preferential applying of RCT in treating ATC patients. We investigated both the OS and CSS because OS has the least methodological issues, consistent with the AJCC publications. However, clinicians and patients are commonly more interested in CSS despite the potential obstacle in reliably determining the cause of death (21).

Overall, the median OS of patients undergoing RCT was twice that of those undergoing RT alone (4 *vs*. 2 months). IPW-adjusted multivariate regressions show the adjusted HR and sHR of RCT *versus* RT alone were 0.69 (95% CI = 0.56–0.85) and 0.77 (95% CI = 0.61–0.96) for OS and CSS, respectively. A similar positive effect of RCT *versus* RT alone was also present within each subgroup by surgical resection and distant metastasis. Notably, the superior role of RCT compared with RT in ATC with distant metastasis is especially promising and of great clinical importance.

The beneficial effect of chemotherapy was found in a study with 79 ATC patients and another study based on the National Cancer Database (12, 22). However, some other small studies failed to identify the positive role of chemotherapy in ATC patients (10, 11). Given the limited sample size of the previous studies, multivariate regression carried out within subsets of ATC patients was unfeasible, such that imbalanced confounding factors may bias their results. A study showed that weekly paclitaxel administration results in significantly prolonged survival but not conventional chemotherapy using doxorubicin or cisplatin (23). However, a study including 100 ATC patients found that any chemotherapy regimen was associated with more prolonged survival (15).

Despite novel therapy, including new regimens and timely intense multimodal treatment, advancement in treating ATC has been very limited. Thus, there is a continuing need to develop more treatment patterns (24). Under the condition of limited treatments, the extended applying of traditional treatment is of great clinical significance. Previous studies have indicated the potential role of chemotherapy in ATC patients but have not comprehensively compared RCT with RT alone. Our study comprehensively compared RCT with RT and suggested the beneficial effect of RCT regardless of surgical resection and distant metastasis. However, confirmatory studies, phase II or possibly phase III studies, are still required and must be designed to define the role of RCT and RT in ATC patients. This is because after the landmark analysis to adjust for potential immortal time bias, the consistently beneficial effect of RCT relative to RT was only seen within ATC patients without distant metastasis. In contrast, the beneficial effects for other subgroups lost statistical significance slightly, although that might be led to by the smaller sample size after landmark analysis that sacrificed samples considerably. Moreover, It is worth mentioning that landmark analysis could also disregard the short-time survival benefit from RCT, particularly for this extremely aggressive cancer (20, 25).

Currently, the most frequently used chemotherapy agents include those against cell division machinery (taxane, paclitaxel, or docetaxel), against DNA repair pathways (anthracycline or doxorubicin), and against DNA structure (cisplatin or carboplatin) (15, 26). Moreover, the most recommended application of cytotoxic chemotherapy is taxane monotherapy in combination with anthracyclines or platin if necessary (26).

Consistent with previous studies, total thyroidectomy provides the best survival outcome compared with nonthyroidectomy and nonsurgical resection in this study (4, 14). An explanation for that is that more aggressive surgery types could contribute to better local control. Distant metastasis was an adverse prognostic factor of survival in our study, consistent with the previous studies (10, 16, 22). Moreover, we found that RCT produces improved survival for both distant metastatic ATC and nondistant metastatic ATC, highlighting the importance of RCT. In our study, the continuous growth of tumors was also explored and controlled. The results show that involving the thyroid cartilage, carotid artery (encased), jugular vein and thyroid artery or vein, bone, skeletal muscle (other than the strap or sternocleidomastoid muscle), mediastinal tissues, and prevertebral fascia was associated with worse survival. Clinicians should carefully consider the continuous growth of ATC.

Interestingly, we failed to find the association of lymph node invasion (AJCC N stage) with OS and CSS, just like a previous study (14). A possible explanation for that may lie in that the extremely short survival of ATC patients leads to the statistical insignificance of lymph node invasion for survival. Similarly, no association of tumor size with survival was found in our study, in line with a small study (16) but contrary to two previous studies presenting a negative correlation of tumor size with survival (14, 27). This discrepancy could be attributed to inadequate confounding factors controlled and small sample size.

At present, there have been no more efficient treatments developed for ATC patients, except for BRAF V600E-mutated ATC that could highly respond to dabrafenib plus trametinib and have a promising outcome (9, 26). A recent study suggested a most promising result of 1 year OS of 94% when applying dabrafenib plus trametinib to BRAF V600E-mutated ATC patients (2). Nevertheless, for ATC without BRAF V600E mutation, timely multimodal and multidisciplinary treatment within high-volume expertise organizations is still the best treatment approach. Our study adds to the evidence of preferentially applying RCT *versus* RT to ATC patients regardless of surgical resection and distant metastasis.

This study has some limitations. (1) This study covered so long a period from 2004 to 2015 that missing factors may bias our findings. Although the year of diagnosis was divided into two intervals at the year 2010 and controlled in multivariate regression. (2) As the nature of the retrospective study, there might be missing confounders that may be important for analysis, which would lead to bias in our research. For example, we did not know the detailed location and the margin status after surgical resection, even though margin status was not associated with survival due to the extreme dismal prognosis of ATC (28). (3) Although variables between groups were balanced using SIPW, the unbalanced confounders of older age, multifocality, metastasis, and tumor extension might still affect the results of this study. Moreover, unmeasured confounding factors may still bias our results. (4) The detailed regimens and doses of chemotherapy were not available in the SEER database, so we could not further explore them. (5) The protocol of radiotherapy referring to dose, fraction size, frequency, and duration was not recorded by the SEER database; thus, the total dose of radiotherapy that could have a tremendous impact on survival was not available and controlled, although the so considered palliative volume was found to be superior to no radiation (22).

Although our study has some limitations, it is the first large retrospective study comprehensively investigating the superior effectiveness of RCT *versus* RT within subsets of ATC patients. Our study suggests the beneficial role of RCT for ATC. Our findings will give support to clinicians to preferentially perform RCT in ATC patients.

## Conclusions

RCT results in significantly prolonged survival in ATC patients, regardless of surgical resection and distant metastasis. RCT should be preferentially performed in ATC patients. Further prospective trials with chemotherapy regimens and radiotherapy doses controlled are needed.

## Data Availability Statement

Publicly available datasets were analyzed in this study. These data can be found here: https://seer.cancer.gov/.

## Ethics Statement

Ethical review and approval was not required for the study on human participants in accordance with the local legislation and institutional requirements. Written informed consent for participation was not required for this study in accordance with the national legislation and the institutional requirements.

## Author Contributions

WZ: Data curation, formal analyses, writing—original draft preparation, and writing—reviewing and editing. YY: Conceptualization, formal analyses, methodology, software, supervision, visualization, writing—original draft preparation, and writing—reviewing and editing. XZ: Conceptualization, data curation, formal analyses, supervision, writing—original draft preparation, and writing—reviewing and editing. All authors contributed to the article and approved the submitted version.

## Conflict of Interest

The authors declare that the research was conducted in the absence of any commercial or financial relationships that could be construed as a potential conflict of interest.

## Publisher’s Note

All claims expressed in this article are solely those of the authors and do not necessarily represent those of their affiliated organizations, or those of the publisher, the editors and the reviewers. Any product that may be evaluated in this article, or claim that may be made by its manufacturer, is not guaranteed or endorsed by the publisher.
